# European Antibiotic Awareness Day 2017: training the next generation of health care professionals in antibiotic stewardship

**DOI:** 10.1007/s00431-017-3055-0

**Published:** 2017-12-05

**Authors:** Lenneke Schrier, Adamos Hadjipanayis, Stefano del Torso, Tom Stiris, Marieke Emonts, Hans Juergen Dornbusch

**Affiliations:** 1 European Academy of Paediatrics, Brussels, Belgium; 2 Willem-Alexancer Children’s Hospital, Leiden University Medical Center, Leiden, The Netherlands; 3 Paediatric Department, Larnaca General Hospital, Larnaca, Cyprus; 4 European University Medical School, Nicosia, Cyprus; 5 Pediatra di Famiglia, Padua, Italy; 6 Department of Neonatal Intensive Care, Oslo University Hospital, Oslo, Norway; 7 Institute of Cellular Medicine, Newcastle University, Newcastle upon Tyne, UK; 8 Department of Paediatric Immunology and Infectious Diseases, Newcastle upon Tyne, UK; 9 Hospital Foundation Trust, Great North Children’s Hospital, Newcastle upon Tyne, UK; 10 Medical University of Graz, Graz, Austria

**Keywords:** Antimicrobial resistance, Postdoctoral training, Paediatrics, Antibiotic stewardship, Antibiotics

## Abstract

Antimicrobial stewardship (AMS) aims to optimise treatment, minimise the risk of adverse effects and reduce health care costs. In addition, it is recognised as a key component to stop the current spread of antimicrobial resistance in Europe. Educational programmes are particularly important for the successful implementation of AMS. Training should start during medical school, continue during clinical training and be reinforced throughout postgraduate training. National core curricula for paediatric training should include passive and active training of competencies needed for AMS and future paediatricians should be skilled in taking leadership roles in AMS initiatives. Other core members of the paediatric AMS team should also receive training focused on the unique medical needs of the paediatric patient.

*Conclusion*: Ideally, all communities, hospitals and health regions in Europe should have AMS that serve all patient types, including children. We all have the responsibility to ensure that existing antibiotics remain effective.

**Table:** 

**What is Known**:
• *Antimicrobial stewardship (AMS) is a key component to stop the current spread of antimicrobial resistance* • *Educational programmes are particularly important for the successful implementation of AMS*
**What is New:** • *All medical doctors in Europe who will be undertaking significant practice in child health should master the competencies needed to prescribe antibiotics to children rationally as described in the European Academy of Paediatrics (EAP) Curriculum for Common Trunk Training in Paediatrics* • *Interdisciplinary approaches of education need to be developed, as all hospitals and health regions in Europe ideally should have AMS programmes that serve all patient types, including children*

## Introduction

Antimicrobial resistance (AMR) poses a serious and increasing threat to public health as infections caused by bacteria that are resistant to antimicrobials lead to approximately 25,000 deaths in the European Union every year [7]. In children, a significant increase in the prevalence of multidrug-resistant bacterial infections has occurred during the past couple of decades [5]. Over the last few years, there have been significant shortages in the development and availability of new antibiotics, and the number of targeted studies on antibiotics in children remains strikingly low [23]. Therefore, the implementation of strategies to preserve the efficacy of existing antibiotics is an urgent public health priority, both in hospital and community settings.

Antimicrobial stewardship (AMS) aims to appropriately and safely prescribe antibiotics to patients, while reducing unnecessary or suboptimal use of antibiotics, thus maximising outcomes for the patient [2]. AMS is recognised as a key component to stop the current spread of AMR in Europe. AMS programmes have the ultimate goal of minimising selective pressure on the emergence of drug-resistant strains. Successful AMS programmes are characterised by the ability to break down activities into specific interventions that can be more easily implemented, monitored and evaluated [3]. Successfully implemented AMS programmes have a significant impact on reducing antimicrobial use in paediatric patients, costs and prescribing errors without negative impacts on patient safety, and actually resulting in improved patient outcomes [13, 23, 24]. The identification of paediatric conditions with both frequent and variable antimicrobial use could guide the prioritisation of high-impact targets for AMS interventions [10]. Paediatric AMS should not be limited to the hospital setting and collaboration among hospital and outpatient health care facilities is of paramount importance [12, 14]. Indeed, AMS can be effective in reducing antibiotic misuse in community settings [9, 11, 26], and potential strategies to promote these programmes in community-based settings have been published [14].

## Training in antimicrobial stewardship

As active participation of clinical professionals at all levels of care is required for AMS to succeed, educational programmes are particularly important for the successful implementation of AMS [25]. Education of health care providers on appropriate antibiotic prescribing has been shown to enhance other antimicrobial stewardship interventions [22]. Therefore, the European Academy of Paediatrics (EAP) Curriculum for Common Trunk Training in Paediatrics [6] includes specific knowledge and skills related to AMR and the European Society for Paediatric Infectious Diseases (ESPID) and other national (paediatric) infectious diseases groups run specific courses on AMR (Table 2). The syllabus for Core Training sets out a European road map for common paediatric training. It is intended as a guide for national paediatric societies to help them understand the principles of core training. The EAP recognises that in many countries in Europe, training does not conform to these recommendations, but as such differences gradually diminish, quality, content and assessment of training, including training related to the principles AMR, will become more uniform across Europe. At the same time, it is recognised that differences in regional epidemiology and healthcare infrastructure require a tailor-made approach, also for AMS. Recently, recommendations were published for key points to be included in clinical curricula, in order to develop the necessary skills to participate in AMS [20, 22]. These are summarised in Table 1. It is important that assimilation of knowledge about AMS starts already during medical school, is continued during clinical training and is reinforced throughout postgraduate training. Passive educational techniques like large and small group presentations are modestly effective for increasing knowledge. However, interactive or dynamic techniques influence prescribing behaviour. This includes education associated with specific episodes of patient care. Interactive small group sessions, e-learning, educational outreach, periodic retrospective audit and feedback and one-on-one patient-directed education have been shown to be moderately to highly effective in optimising antibiotic use and patient outcomes [22]. In particular, internet-based education shows encouraging results [15, 17, 18]. The advantages of e-learning include improved access to education in rural or low-resource areas and the potential to develop an interactive platform. Several examples of e-learning tools are listed in Table 2. In addition, AMS was recently taught during the EAP MasterCourse 2017 and is included in the upcoming European Academy of Paediatric Societies congress in Paris, 2018. In addition, AMS is always covered during the annual residential ESPID-Oxford Course.

**Table 1:** Key points to include in clinical AMS curricula [adapted from [20, 22]

	Learning objective(s)	Topics
Effect of AMS initiatives on clinical outcome	The participant learns about how AMS contributes to accurate and safer prescribing of antimicrobials, resulting in improved clinical outcomes	Effective treatment
		Clinical outcomes (mortality and morbidity)
		Reduction of side effects
		Population specific approaches
Effect of AMS initiatives on bacterial resistance	The participant learns about antibiotic resistance mechanisms, including causes and extent	Epidemiology (global)
		Genetics and mechanisms
		Relationship to antibiotic use
		Discussion of AMS initiatives like prospective audit with feedback and formulary restriction
	The participant learns how AMS reduces the spread of antimicrobial resistance	
Diagnosis of infection	The participant learns how to accurately interpret laboratory reports in order to make clinical treatment decisions in neonates, infants, children and adolescents;	Proper use and interpretation of bacterial Gram stain/culture, rapid and point-of-care tests, serology, and biomarkers of infection
		Establishment of standardised diagnostic criteria for specific infections
	The participant learns how to diagnose an infection in a standardised manner;	
Principles of infection management in children	The participant learns how to make informed treatment decisions early in the course of disease in order to positively influence treatment outcomes;	Promptly identify patients who require antibiotics
		Timely and appropriate initiation of antibiotics
		Obtain cultures before starting antibiotics
		Do not give antibiotics with overlapping activity or combinations not supported by evidence or guidelines
	The participant learns how to de-escalate antibiotic use in order to more effectively treat an infection while limiting exposure to broad-spectrum antimicrobials	
		Determine and verify antibiotic allergies
		Consider local antibiotic susceptibility
		Specify expected duration of therapy based on evidence and national and hospital guidelines
		Ensure appropriate administration (intravenous versus oral)
		Give antibiotics at the right dose and interval
		Stop or de-escalate therapy promptly based on the culture and sensitivity results or establishment of an alternative diagnosis
		Reconcile and adjust antibiotics at all transitions and changes in patient’s condition
		Monitor for toxicity reliably and adjust agent and dose promptly
Prescribing of antibiotics	The participant learns the basics needed to prescribe antibiotics for infections caused by susceptible and resistant organisms	Pharmacokinetics and mechanism of action of different classes of antibiotics (‘bug-drug’ coverage)
		Pharmacology and adverse effects, including risk of *C. difficile* infection
		Principles of empirical versus directed therapy
		Drug purchasing and dispensing costs
Guidelines for diagnosis and management of most frequent infections in children		Specific instruction for these common infections using principles outlined above
		Use of national and local guidelines and public health guidance
Prevention of infection	The participant learns about the importance of preventive measures to limit the development of antimicrobial resistance	Hand hygiene
		Prudent use of catheters and devices
		Principles and duration of surgical prophylaxis
Communication skills	The participant learns how to apply communication techniques to talk with patients and families about prudent antibiotic use

**Table 2:** Available e-learning tools

AMS (general)	Collection of AMS online courses made by the European Centre for Disease Prevention and Control: https://ecdc.europa.eu/en/publications-data/directory-guidance-prevention-and-control/training-antimicrobial-stewardship Massive Open Online Course on Antimicrobial Stewardship (University of Dundee, UK): https://www.futurelearn.com/courses/antimicrobial-stewardship Antimicrobial Stewardship Online CME Courses (Stanford University School of Medicine, USA): https://med.stanford.edu/cme/learning-opportunities/antimicrobialstewardship.html
Paediatric AMS	European Society for Paediatric Infectious Diseases (ESPID) online course on paediatric AMS: http://www.espid.org/content.aspx?Page=ESPID%20Online%20Antibiotic%20Management%20Course

Although many of the overarching principles of AMS apply to children and adults alike, many factors related to paediatric AMS are unique to children. Children have high rates of infection and frequently present with non-specific symptoms adding to diagnostic uncertainty. Patterns of infection and resistance vary significantly by age, thus age-specific antibiotic panels for antibiograms should preferably be used to guide antibiotic choices for selected infections. Children are more prone to infection with resistant organisms due to a future lifetime of antibiotic exposure. In addition, considerations related to age-appropriate dosing and formulations pose challenges to the prescription of antibiotics in children [4]. Children may respond differently to antibiotics compared to adults. Finally, immunisation initiatives should be included in AMS programmes as a preventive strategy in both in- and outpatient paediatric settings, in order to decrease the likelihood of serious illness and to decrease AMR. Therefore, other core members of the paediatric AMS team, like the microbiologist, paediatric infectious disease specialist and clinical pharmacist, should receive training focused on the unique medical needs of the paediatric patient [16, 19]. In addition, future paediatricians should be skilled in taking leadership roles in AMS initiatives and develop practical solutions rooted in the general principles of AMS, as it is important to further expand AMS activities from the hospital to paediatric offices and communities [14].

## Keep antibiotics working

Limiting the further spread of AMR is one of EAP’s child health priorities for 2017–2018. We will work together with the European Centre for Disease Prevention and Control to raise awareness about the relevance and benefits of cost-effective AMS policies [21] and training in child health and to advocate for sustained implementation of these across Europe. Europe would greatly benefit from a uniform adoption of AMR and infection prevention best practice across countries. This includes harmonisation and simplification of the various treatment guidelines that currently exist across Europe. Moreover, there is an urgent need to conduct research on new antibiotic (classes) for critical multi-drug-resistant pathogens and on the effects of AMS programmes in low-income countries in which emerging resistance are particularly alarming [1]. In addition, limited access to high-quality antibiotics is particularly of concern in these countries. Continuous education and training of health care professionals on appropriate antibiotic use is crucial. We therefore welcome the new European Action Plan, which was launched this summer. The European Commission intends, among others, to develop training programmes on AMR for health professionals through the ECDC and the EU health programme [8].

## Conclusion

Prevention of AMR needs rigorous actions in the community, at practice, ward, institutional, national and international levels. Best practices should be applied cross-border and healthcare institutions and communities should collaborate regionally and internationally, in order to fight AMR successfully. Effective Europe-wide implementation and sustained use of cost-effective antimicrobial policies can consequently lead to improved safety and quality of care while contributing to more sustainable healthcare. Ideally, all communities, hospitals and health regions in Europe should have AMS programmes that serve all patient types, including children. This includes adult academic and community hospitals and outpatient care centres that primarily care for children. Therefore, the EAP is interested to discuss interdisciplinary approaches of education with other stakeholders.

Prudent use of antibiotics is very important to address the global challenges posed by AMR. We all have the responsibility to ensure that existing antibiotics remain effective.

## Abbreviations

AMR: Antimicrobial resistance
AMS: Antimicrobial stewardship
CRP: C-reactive protein
EAP: European Academy of Paediatrics
ESPID: European Society for Paediatric Infectious Diseases
